# Human amniotic mesenchymal stem cells and their paracrine factors promote wound healing by inhibiting heat stress-induced skin cell apoptosis and enhancing their proliferation through activating PI3K/AKT signaling pathway

**DOI:** 10.1186/s13287-019-1366-y

**Published:** 2019-08-09

**Authors:** Jing-Yuan Li, Kang-Kang Ren, Wen-Jie Zhang, Ling Xiao, Han-You Wu, Qian-Yu Liu, Ting Ding, Xiang-Cheng Zhang, Wen-Jia Nie, Yu Ke, Ke-Yu Deng, Quan-Wen Liu, Hong-Bo Xin

**Affiliations:** 1 The National Engineering Research Center for Bioengineering Drugs and the Technologies, Institute of Translational Medicine, Nanchang University, No. 1299 Xuefu Road, Honggutan District, Nanchang, 330031 People’s Republic of China; 2 School of Life and Science, Nanchang University, Nanchang, 330031 People’s Republic of China; 3 Department of Obstetrics and Gynecology, The First Affiliated Hospital of Nanchang University, Nanchang, 330006 People’s Republic of China

**Keywords:** Human amniotic membrane mesenchymal stem cells, Conditioned medium, Wound healing, PI3K/AKT signaling, Antibody array

## Abstract

**Background:**

Increasing evidence has shown that mesenchymal stem cells (MSCs) yield a favorable therapeutic benefit for thermal burn skin wounds. Human amniotic MSCs (hAMSCs) derived from amniotic membrane have multilineage differentiation, immunosuppressive, and anti-inflammatory potential which makes them suitable for treating skin wounds. However, the exact effects of hAMSCs on the healing of thermal burn skin wounds and their potential mechanisms are not explored.

**Methods:**

hAMSCs were isolated from amniotic membrane and characterized by RT-PCR, flow cytometry, immunofluorescence, and tumorigenicity test. We assessed the effects of hAMSCs and hAMSC conditional medium (CM) on wound healing in a deep second-degree burn injury model of mice. We then investigated the biological effects of hAMSCs and hAMSC-CM on the apoptosis and proliferation of heat stress-injured human keratinocytes HaCAT and dermal fibroblasts (DFL) both in vivo and in vitro. Next, we explored the underlying mechanisms by assessing PI3K/AKT and GSK3β/β-catenin signaling pathways in heat injured HaCAT and DFL cells after hAMSCs and hAMSC-CM treatments using PI3K inhibitor LY294002 and β-catenin inhibitor ICG001. Antibody array assay was used to identify the cytokines secreted by hAMSCs that may activate PI3K/AKT signaling pathway.

**Results:**

Our results showed that hAMSCs expressed various markers of embryonic stem cells and mesenchymal stem cells and have low immunogenicity and no tumorigenicity. hAMSC and hAMSC-CM transplantation significantly promoted thermal burn wound healing by accelerating re-epithelialization with increased expression of CK19 and PCNA in vivo. hAMSCs and hAMSC-CM markedly inhibited heat stress-induced apoptosis in HaCAT and DFL cells in vitro through activation of PI3K/AKT signaling and promoted their proliferation by activating GSK3β/β-catenin signaling. Furthermore, we demonstrated that hAMSC-mediated activation of GSK3β/β-catenin signaling was dependent on PI3K/AKT signaling pathway. Antibody array assay showed that a panel of cytokines including PAI-1, C-GSF, periostin, and TIMP-1 delivered from hAMSCs may contribute to the improvement of the wound healing through activating PI3K/AKT signaling pathway.

**Conclusion:**

Our results demonstrated that hAMSCs and hAMSC-CM efficiently cure heat stress-induced skin injury by inhibiting apoptosis of skin cells and promoting their proliferation through activating PI3K/AKT signaling pathway, suggesting that hAMSCs and hAMSC-CM may provide an alternative therapeutic approach for the treatment of skin injury.

**Electronic supplementary material:**

The online version of this article (10.1186/s13287-019-1366-y) contains supplementary material, which is available to authorized users.

## Background

Skin is considered one of the most vital organs in the body due to its important functions such as an outer protective barrier against various external agents and a temperature regulator [1]. The serious consequences of cutaneous wound, both acute and chronic, can be caused by many different factors. Cutaneous wound healing requires well-coordinated responses of inflammation, cell proliferation, neovascularization, extracellular matrix formation, and re-epithelialization [2, 3]. With a high rate of morbidity and mortality, skin burn is not only difficult to treat, but also poses a major public health burden worldwide [4]. Thermal burns can be caused by dry sources (fire or flame) and wet sources (scalds) and classified based on the depth of burn [5]. Compared to other wounds, thermal burn wounds are characterized by delayed wound healing because of edema, bacterial infection, chronic inflammation and necrosis [6]. Lack of autologous skin sources or immunological rejection of allogeneic skin brings about the dilemma of clinic treatment of thermal burn [7]. Therefore, developing new and effective wound healing therapies, especially for thermal burn wounds, is urgent.

Recently, stem cell therapy, especially using MSCs, has emerged as a promising new and effective therapeutic strategy for accelerating cutaneous wound healing [2], and this protective effect was predominantly mediated by paracrine rather than direct regenerative mechanisms [8]. Numerous studies have shown that MSCs promote cutaneous wound healing by accelerating wound closure [9, 10], enhancing angiogenesis [11, 12], inhibiting the inflammatory response [13, 14], regulating extracellular matrix remodeling [15, 16], inhibiting cell apoptosis, and promoting cell proliferation [17]. Meanwhile, MSCs have been studied as one of the more promising therapies for the healing of the thermal burns [7, 18–20]. Recently, human amniotic membrane-derived mesenchymal stem cells (hAMSCs) have been recognized as one of the most promising stem cells in the field of regenerative medicine. The active proliferative potential, low immunogenic profile, anti-inflammatory function [21], and tissue repair ability of hAMSCs can be beneficial to the burn wound healing as well as the severe systemic effects of burn, including hypermetabolic response, inflammation-related diseases, and immunosuppression [22]. Anna et al. have previously found that conditioned medium derived from MSCs could enhance normal skin fibroblast proliferation and migration and promote wound healing in an excisional full-thickness skin murine model [15]. Similarly, MSC-derived exosomes have been proved to contribute to thermal burn wound healing [17]. Therefore, it is suggested that the effect of MSCs on the wound healing was mediated via a paracrine signaling mechanism. However, the exact effects of hAMSCs on the thermal burn wound healing and their potential mechanism are not explored.

In the present study, we isolated hAMSCs from human amniotic membrane and characterized their morphology, phenotypic profiles, pluripotency, tumorigenicity, and growth potency. In vivo, we investigated the role of hAMSCs in thermal burn wound healing using a mouse deep second-degree burn injury model. We found that hAMSC and hAMSC-CM transplantation promoted skin wound healing by enhancing proliferation and inhibiting apoptosis of skin cells in the wound area. In vitro, a transwell co-culture system and hAMSC-CM were used to assess the influences of hAMSC-secreted factors on the apoptosis, proliferation, and migration of heat injury skin cells. The results showed that hAMSCs or hAMSC-CM inhibited heat stress-induced apoptosis in HaCAT and DFL cells and promoted their proliferation through activation of PI3K/AKT signaling pathway with a paracrine manner. In addition, antibody array assay showed that hAMSCs could deliver many cytokines including PAI-1, C-GSF, periostin, TIMP-1, uPAR, and so on, which may activate PI3K/AKT signaling pathway.

## Materials and methods

### Isolation, culture, and expansion of hAMSC_S_

Human fetal placentas were obtained from the Department of Obstetrics and Gynecology, The First Affiliated Hospital of Nanchang University. The verbal consent was obtained from all of the volunteers prior to their participation. The research procedure was approved by the ethics committee of The First Affiliated Hospital of Nanchang University. The amnion is a thin, avascular membrane composed of human amniotic epithelial stem cells (hAESCs) and hAMSCs. For isolation of hAMSCs, hAESCs were firstly released from the amniotic membrane as previously described [21]. Then, the amnion was washed three times with HBSS and digested with Collagenase IV(1 g/L, Thermo Fisher, Nanchang, China) on a rotator 40 min at 37 °C. Digestion was terminated by addition of medium containing 10% FBS; the single-cell suspension was filtered through a 70-μm cell strainer (BD Labware, Shanghai, China) and centrifuged at 1000 rpm for 5 min. The supernatant was discarded, and the cells were re-suspended with α-MEM medium (Thermo Fisher) containing 18% Chang B, 2% Chang C (Irvine Scientific), 10% FBS, 1% glutamine, and 1% penicillin/streptomycin (Gibco). hAMSCs were placed in cell culture dishes (Corning, NY, USA) at a density of 5 × 10^4^ cells/cm^2^ at 37 °C with 5% CO_2_ atmosphere. Unattached cells and debris were removed after 2 days. In each experiment, the cells were grown to approximately 80% confluence, and only cells between passages 3 and 7 were used for subsequent experiments.

### Collection of conditioned medium (CM) of hAMSC_S_

For the collection of hAMSC-CM, hAMSCs were grown in a normal culture medium. Once the cells reached 80% confluency, the medium was changed to high-glucose Dulbecco’s modified Eagle’s medium (H-DMEM, Thermo Fisher) containing 100 U/ml penicillin/streptomycin. CM was collected after 48 h and centrifuged at 1500 rpm for 5 min to ensure complete removal of cellular debris. CM was then concentrated 10-fold by using an Amicon® Ultra 3 K device (MilliporeSigma, USA).

### Reverse transcription-polymerase chain reaction (RT-PCR)

Total RNA from each sample was extracted by the Trizol reagent (Thermo Fisher). Purity was assessed by the absorbance ratio at 260 and 280 nm. RNA (100 ng to 1 μg) was reverse transcribed into cDNA with the M-MLV Reverse Transcriptase (Promega, Shanghai, China) according to the manufacturer’s instructions. The primers for the target products were designed as in Table 1. Polymerase chain reactions (PCR) were carried out in a PCR thermal cycler (Thermo Hybaid, Waltham, MA, USA). PCR products were electrophoresed on a 1.0% (m/v) agarose gel containing 0.5 μg/ml ethidium bromide for nucleic acid visualization under UV light. In parallel, mRNA levels of human housekeeping GAPDH were analyzed as an internal normalization control.

**Table 1:** Primers and conditions used for RT-PCR to detect gene transcripts in hAMSCs

Genes	Sequence	TM (°C)	Size (bp)
GAPDH	F:5′-CCACCCATGGCAAATTCCATGGCA-3′	59	598
	R:5′-TCTAGACGGCAGGTCAGGTCCACC-3′		
Nanog	F:5′-CAATGGTGTGACGCAGGGAT-3′	52	149
	R:5′-TGCACCAGGTCTGAGTGTTC-3′		
OCT4	F:5′-ATCCCTGAACCTAGTGGGGA-3′	59	480
	R:5′-CACTCGGACCACATCCTTCT-3′		
CD105	F:5′-TCCTCCCAAGGACACTTGTA-3′	57	244
	R:5′-CGCCTCATTGCTGATCATAC-3′		
CD29	F:5′-CCGCGCGGAAAAGATGAAT-3′	55	406
	R:5′-AAATGTCTGTGGCTCCCCTG-3′		
CD90	F:5′-GAGGGAGGAAGAGCAGACCT-3′	57	896
	R:5′-CCTGGATCGGGTTATGATGGG-3′		
CD34	F:5′-GCAAGCCACCAGAGCTATTC-3′	55	390
	R:5′-GGTCCCAGGTCCTGAGCTAT-3′		
CD133	F:5′-ATCCTTTCCATTACGGCGGC-3′	57	311
	R:5′-CTCAAGGCACCATCCCGTG-3′		

*F* Forward primer, *R* Reverse primer

### Identification of hAMSCs by flow cytometry

Phenotypic analyses of cultured hAMSCs were performed using standard flow cytometry methods. Passage 3 hAMSCs were collected in fluorescence-activated cell sorting (FACS) tubes (BD Biosciences, Franklin Lakes, NJ) at a concentration of 1 × 10^6^ cells/ml in stain FACS buffer (PBS containing 2% FBS) and then stained with FITC-conjugated antibodies against human CD29, CD90, CD45, HLA-DR, CD80, and CD40; phycoerythrin (PE)-conjugated antibodies against human CD73, CD105, CD34, HLA-ABC, and CD86; and their isotype controls (all from BD Biosciences) at 4 °C for 30 min in the dark. After washing twice, the cells were resuspended in 200 μl of PBS and acquired by a FACSCalibur instrument (BD Biosciences). Data were analyzed using FLOWJO TM software (TreeStar, Inc., Ashland, OR, USA).

### Immunofluorescence

Immunofluorescence experiments were carried out following our previously reported protocols [21]. Briefly, cells growing on the glass slide were fixed with 4% paraformaldehyde for 15 min and permeabilized using 0.25% Triton X-100 diluted in PBS for 10 min at room temperature. To block unspecific epitopes, cells were incubated with PBS containing 1% BSA and 0.1% Tween-20 for 1 h. Cells were then incubated with the following primary antibodies at 4 °C overnight: rabbit anti-OCT4 (5 μg/ml, Abcam, Nanchang, China), mouse anti-SSEA-4 (15 μg/ml, Abcam), rabbit anti-Nanog (1:200, Abcam), rabbit anti-Ki67 (1:100, Abcam), and mouse anti-PCNA (5 μg/ml, Abcam). After that, cells were incubated with secondary donkey anti-mouse or anti-rabbit antibodies conjugated to either Alexa Fluor 488 or Alexa Fluor 568 (Jackson, Nanchang, China). Nuclei were counterstained with DAPI (Thermo Fisher).

### Adipogenic and osteogenic differentiation

Passage 3 hAMSCs were seeded at a density of 1.5 × 10^5^ cells/well in a six-well plate. When the cells reached 100% confluence, OriCell™ human mesenchymal stem cell adipogenic differentiation medium (Cyagen Biosciences, Shanghai, China) was added to wells according to the manufacturer’s instruction. After 24 days of induction, Oil red O (Cyagen Biosciences) staining was performed to assess the differentiation potential of adipogenesis formation of intracellular lipid droplets. For osteogenic differentiation, hAMSCs were cultured with OriCell™ human mesenchymal stem cell osteogenic differentiation medium (Cyagen Biosciences) for 23 days to analyze the osteogenic differentiation. The differentiation potential for osteogenesis was assessed by Alizarin Red (pH 4.2, 40 mM) (Cyagen Biosciences) staining.

### Soft agar tumorigenicity test

Each well of the 6-well culture plates was first coated with 0.6% soft agar (bottom layer). A 0.3% soft agar containing hAMSC_S_ (1 × 10^3^/well) was then layered on top of the 0.6% gel and incubated at 37 °C with 5% CO_2_ for 30 days. Human liver carcinoma cell HepG2 was used as the control. Colony formation was observed and imaged by phase-contrast microscopy.

### In vivo tumorigenicity test

To test the tumorigenicity of hAMSC_S_ in vivo, 5 × 10^6^ hAMSCs (in 200 μl PBS) were injected into the left thigh muscle and the right back of NOD-SCID mice, respectively. The same number of embryonic stem cells was used as positive control. The tumor-forming was monitored every day for up to 20 weeks.

### In vivo skin wound model and treatment

Adult male C57BL/6 mice (8 weeks old) were purchased from Changsha SLAC Laboratory Animal Company (Changsha, China, http://www.hnsja.com/) and housed under standard laboratory conditions with standard chow and water daily at the Laboratory Animal Center of Institute of Translational Medicine of Nanchang University. All animal procedures described here were reviewed and approved by the Animal Care and Use Committee of Nanchang University. Mice were anesthetized, and back cutaneous hair was removed by electrical shaving. The back skin of mice was injured with 80 °C water for 100 s to create a 10-mm diameter wound. Meanwhile, 200 μl PBS, 2 × 10^6^ hAMSCs suspended in 200 μl PBS, 200 μl H-DMEM (10X), or 200 μl hAMSC-CM (10X) were injected subcutaneously near the wound at four sites. The normal group had no treatment.

### Whole-body fluorescent imaging

For the purpose of cell tracking, hAMSCs were labeled with PKH26 red fluorescent dye (Sigma-Aldrich) and then injected subcutaneously near the wound at four sites. Mice were anesthetized after 0 days, 7 days, 14 days, and 21 days of cell injection and visualized with whole-body fluorescent imaging system (LB983; Berthold, Germany).

### Histopathology and TUNEL assay

Skin tissue samples of all groups were excised and fixed in 4% paraformaldehyde, embedded in paraffin, sectioned at 5-μm thickness, and mounted on slides. The slides were deparaffinized and stained with hematoxylin and eosin (H&E), PCNA (1:1000, mouse monoclonal, Abcam), CK19 (1:1000, mouse monoclonal, Abcam), Anti-Human Nuclei Antibody MAB1231 (1:200, mouse monoclonal, Merck), CD90 (1:250, rabbit monoclonal, Abcam), and CD31 (1:100, mouse monoclonal, Abcam).

Apoptosis was analyzed on paraffinic skin tissue sections of different group by TUNEL assay kit (Millipore, USA). Three sections were selected for each mouse and stained using the TUNEL assay kit following the manufacturer’s protocol.

### Western blot analysis

Total protein was extracted from HaCAT cells, DFL cells, and skin tissues. Western blot was performed to detect the target proteins. Sixty micrograms of total protein was run on 10% denaturing SDS-PAGE gels, then transferred to nitrocellulose membranes (BioRad), which were incubated with primary antibodies anti-GAPDH (1:1000, rabbit monoclonal, Santa Cruz), anti-β-actin (1:1000, mouse polyclonal, CST), anti-Bcl-2 (1:1000, mouse monoclonal, Abcam), anti-Bax (1:1000, mouse monoclonal, Abcam), anti-PCNA (1:1000, mouse monoclonal, Abcam), anti-CK19 (1:1000, mouse monoclonal, Abcam), PI3K (1:1000, rabbit polyclonal, CST), P-PI3K (1:1000, rabbit polyclonal, CST), anti-AKT (1:1000, mouse monoclonal, Abcam), anti-P-AKT (1:1000, mouse monoclonal, Abcam), mTOR (1:1000, rabbit polyclonal, CST), P-mTOR (1:1000, rabbit polyclonal, CST), anti-β-Catenin (1:1000, mouse monoclonal, Abcam), anti-GSK3β (1:1000, mouse monoclonal, Abcam), and anti-P-GSK3β (1:1000, mouse monoclonal, Abcam) at 4 °C overnight. Blots were detected with horseradish peroxidase (HRP)-conjugated goat anti-rabbit or rabbit anti-mouse secondary antibody (Invitrogen) for 1 h at room temperature. Images were quantified using the Super Signal West Pico or Femto chemiluminescent detection system (Pierce).

### Tube formation assay

Human umbilical vein endothelial cells (HUVECs) were obtained from ATCC and cultured following the manufacturer’s instructions. Matrigel Basement Membrane Matrix (BD Biosciences, CA, USA) was added in 48-well plates (130 μl per well) and solidified at 37 °C for 1 h. Then, 2.0 × 10^4^ HUVECs per well were seeded and cultured with normal medium or normal medium supplemented with 10% hAMSC-CM (10X). After incubating at 37 °C and 5% CO2 for 2 h, 4 h, and 6 h, the tube formation was detected under a microscope.

### In vitro co-culture experiment

Keratinocytes HaCAT cells were purchased from ATCC and cultured in H-DMEM supplemented with 15% FBS and 1% penicillin/streptomycin (all from Thermo Fisher) at 37 °C in a 5% CO_2_ humidified atmosphere. DFL were isolated from discarded circumcised foreskin on healthy boys as previously described [23, 24] and cultured in H-DMEM containing 15% FBS and 1% penicillin/streptomycin at 37 °C with 5% CO_2_. DFL cells between passages 3 to 5 were used for subsequent experiments.

Skin cells (HaCAT and DFL) were trypsinized and seeded in a 6-well dish at 1.5 × 10^5^ cells/well. To mimic the burn injury model in vivo, cells were treated at 43 °C for 50 min in a water bath. For the normal medium (NM) group, cells were incubated with 3 ml H-DMEM containing 15% FBS and 1% penicillin/streptomycin. For hAMSCs group, a co-culture transwell chamber (2.4-cm diameter, 0.4-μm pore size; Corning) was used to assess the effects of hAMSCs on heat stress-injured skin cells in vitro. Skin cells were seeded into the lower chamber in 2.0 ml of H-DMEM with 15% FBS, and hAMSCs were seeded in the upper compartment at a 1:1 ratio with skin cells in 1.0 ml of the same medium. For the hAMSC-CM group, cells were cultured with 3 ml H-DMEM supplemented with 10% hAMSC-CM (10X), 15% FBS, and 1% penicillin/streptomycin. Skin cells cultured in the normal medium not treated at 43 °C for 50 min were used as the control group. Samples were collected after culturing for 24 and 48 h.

### In vitro skin cells proliferation and apoptosis analysis

Cell proliferation was evaluated at indicated time points using the CCK-8 kit (Dojindo Laboratories, Kumamoto, Japan), following the manufacturer’s protocol. CCK-8 reagent (10%) was added to each well for 3 h at 37 °C. Viability was evaluated by measuring the absorbance at a 450-nm wavelength with using a microplate spectrophotometer (BioRad).

For the apoptosis assays, 1.0 × 10^5^ cells were collected from each sample and resuspended in 100 μl Annexin V binding solution containing 5 μl Annexin V-FITC and 5 μl propidium iodide (PI) solution (Dojindo). After incubation for 15 min at room temperature, cells were washed in PBS, centrifuged at 1000 rpm for 5 min, and resuspended in 400 μl Annexin V Binding Buffer. The apoptosis assays were run and analyzed with BD Jazz.

### In vitro scratch-wound-closure assay

HaCAT and DFL cells were seeded into 6-well dishes at a density of 1.5 × 10^5^ cells per well. When the cells reached 100% confluency, the monolayers were scratched using a sterile 200 μl pipette tip. After disruption, monolayers were gently washed twice with PBS to remove cell debris. Subsequently, the cells were treated with PBS, hAMSCs, or hMBSC-CM. The dishes were incubated at 37 °C in a 5% CO_2_ air atmosphere for 48 h. Images were acquired at 48-h time points, and the migration area of HaCAT and DFL cells were measured by using Image Pro Plus 6.0 software.

### Cytokine antibody array

We collected 3 hAMSC-CM samples; the profiles of cytokines secreted by hAMSCs were detected in the culture supernatants using a Human Cytokine Array (RayBiotech, Guangzhou, China) according to the manufacturer’s instructions.

### Statistical analysis

The results are presented as average value ± standard deviation (SD). Student’s *t* test was used for analysis between two groups. One-way analysis of variance (ANOVA) was used to compare data among three or more groups. Differences with a *P* value of < 0.05 were considered statistically significant.

## Results

### Identification and characterization of hAMSCs

**Fig. 1:**
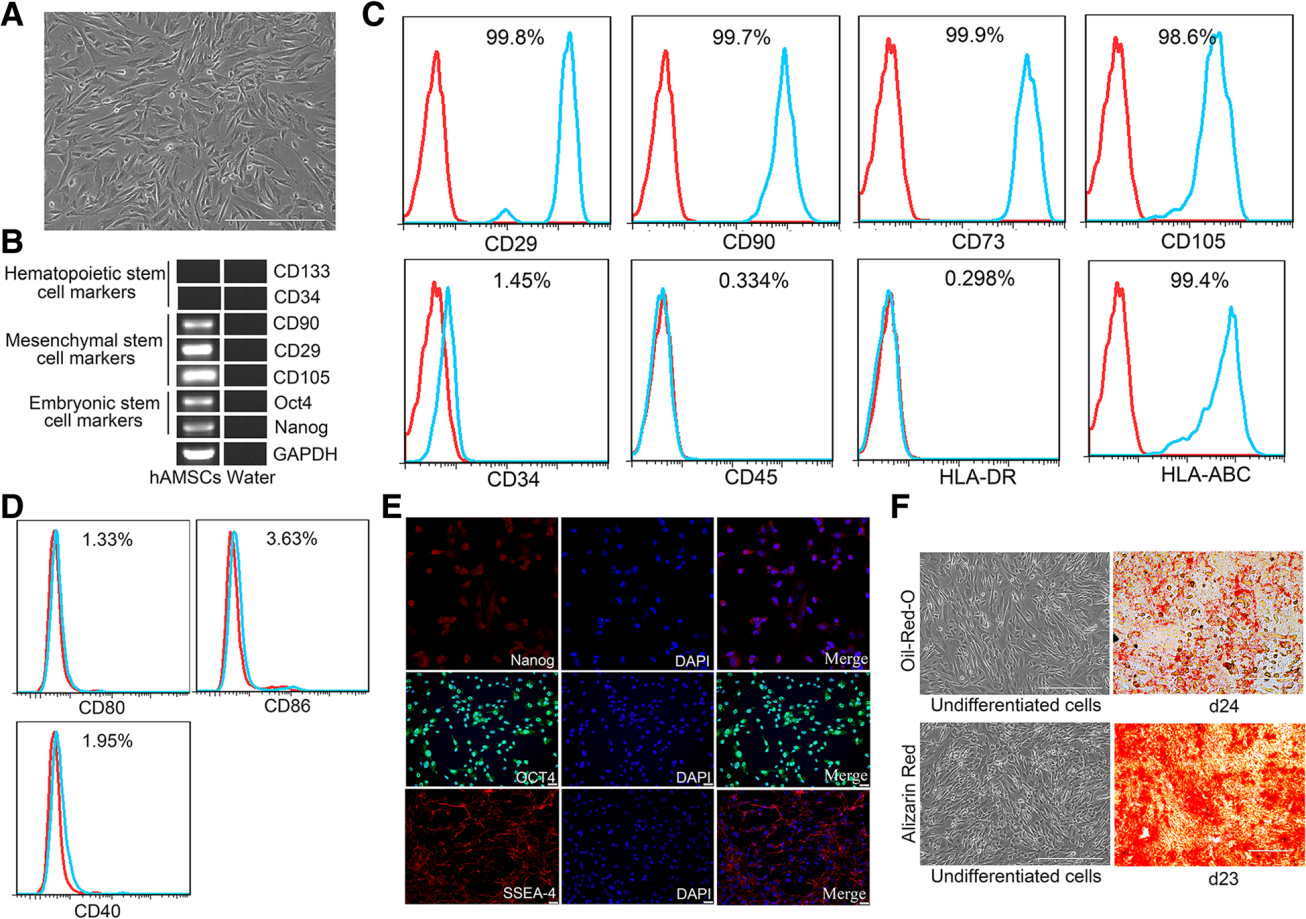
Identification of hAMSCs. **a** Representative photomicrograph of adherent hAMSCs with spindle shapes on plastic cell culture dish. **b** RT-PCR analysis for the expressions of markers in hAMSCs. hAMSCs specifically expressed various markers of Nanog, Oct4, CD105, CD29, and CD90, but not CD34 and CD133. Water was used as negative control. **c** Flow cytometry analysis of CD29, CD90, CD73, CD105, CD34, CD45, HLA-DR, and HLA-ABC expression in hAMSCs. The red lines represent the isotype control, and the blue lines represent the level of surface markers. **d** The hAMSCs were negative for HLA-ABC co-stimulatory molecules CD80, CD86, and CD40. **e** Immunofluorescence staining of the embryonic stem cell surface markers Oct4, SSEA-4, and Nanog in hAMSCs. **f** Multiple differentiation potential of hAMSCs. The hAMSCs were differentiated into matured adipocytes and osteocytes after incubation with adipogenic or osteogenic differentiation medium at the times indicated, respectively. Adipocytes and osteocytes differentiated from hAMSCs were determined by staining with Oil Red O and Alizarin Red, respectively

Cultured primary and passaged hAMSCs formed a monolayer of adherent cells and exhibited a spindle-shaped, fibroblast-like morphology. In the presence of bFGF (10 ng/ml), the hAMSCs were proliferated robustly and the average doubling time was 2 days (Fig. 1a). hAMSCs were positive for mesenchymal stem cell markers CD29, CD73, CD105, and CD90 and negative for hematopoietic stem cell markers CD133, CD34, and CD45 as determined by RT-PCR and flow cytometry (Fig. 1b, c). hAMSCs also expressed the major histocompatibility protein HLA-ABC but none of its co-stimulatory molecules CD80, CD86, and CD40, nor major histocompatibility protein HLA-DR (Fig. 1c, d), indicating that these cells possess low immunogenicity. The expressions of embryonic stem cell surface markers Nanog, Oct4, and SSEA-4 were also analyzed by RT-PCR and immunofluorescence. The results showed that hAMSCs expressed all of these pluripotent markers (Fig. 1b, e), indicating that hAMSCs have the capacity of self-renew as well as multi-lineage differentiation potentials. To identify the multipotency of hAMSCs, we performed adipogenic and osteogenic differentiation assays. Under adipogenic and osteogenic differentiation conditions, most hAMSCs could differentiate into adipocytes and osteocytes, respectively (Fig. 1f).

Soft agar colony formation assay provides an ideal tool for identifying the tumorigenicity of hAMSCs in vitro. After 30 days of cell growth in the soft agar, many colonies were visible in Hepg2 group but no colony was observed in the hAMSCs group at the same time **(**Additional file 1: Figure S1A). In addition, we injected the hAMSCs and embryonic stem cells (positive control) into the left thigh muscle and right back of NOD-SCID mice. The results showed that large tumors were formed in all mice implanted with embryonic stem cells within 8 weeks (*n* = 5). In contrast, no tumor formation in any of the hAMSC-injected animals over a time period of 20 weeks **(**Additional file 1: Figure S1B).

### hAMSC and hAMSC-CM transplantation accelerates skin wound healing in a mouse model

**Fig. 2:**
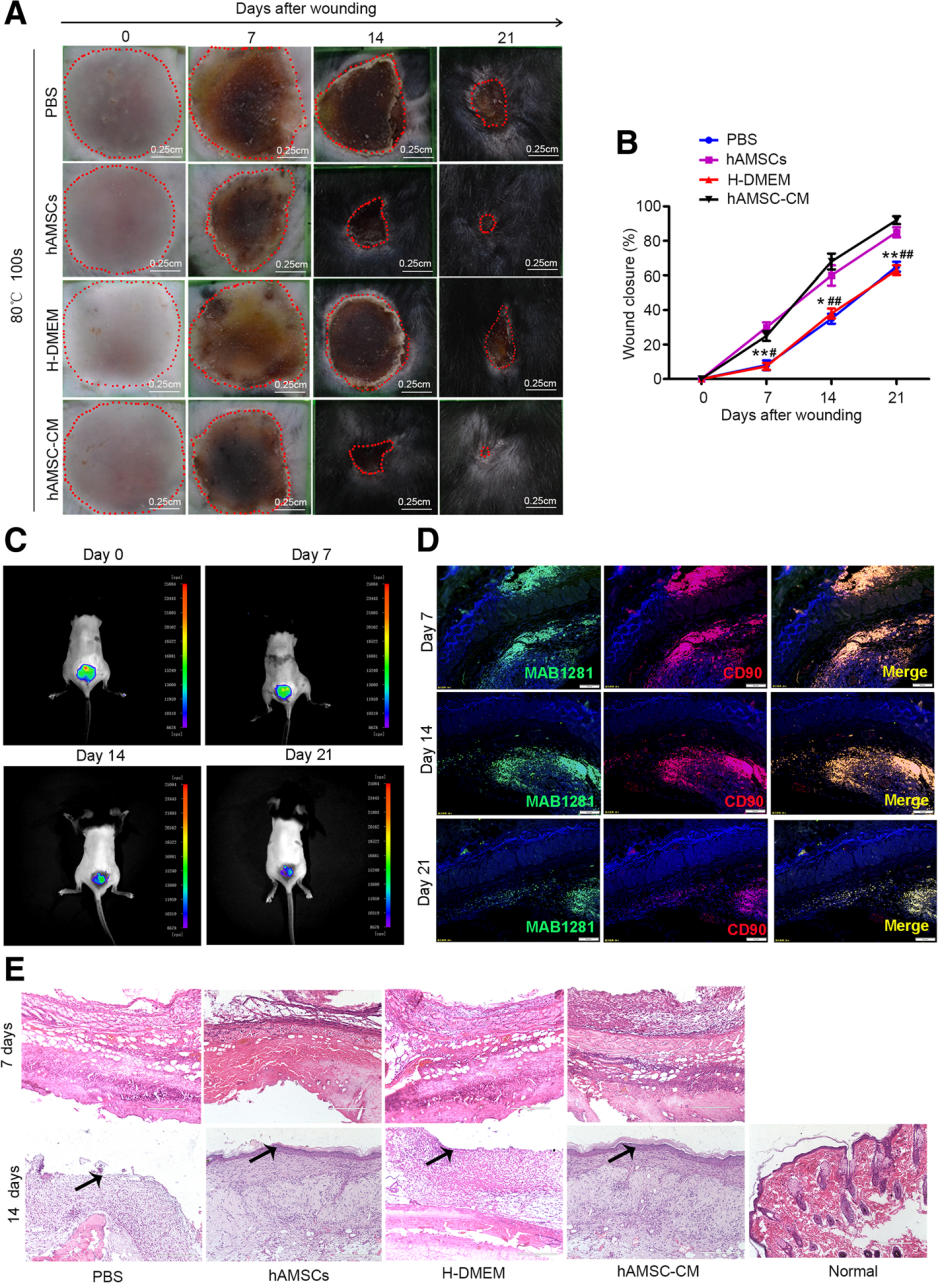
hAMSCs or hAMSC-CM accelerated wound closure by subcutaneously injected around the injured site in C57BL/6 mice. **a** Representative images of wounds after transplantations of PBS, hAMSCs, H-DMEM, and hAMSC-CM at day 0, day 7, day 14, and day 21. **b** Measurement of wound closure at different time points treated with PBS, hAMSCs, H-DMEM, or hAMSC-CM. The results showed that the wound closure was significantly increased in response to hAMSCs and hAMSC-CM (*n* = 5). The percentage of wound closure was calculated as (area of original wound − area of measured wound)/area of original wound × 100. *PBS group compared with hAMSCs group; #H-DMEM group compared with the hAMSC-CM group. **c** Whole-body fluorescent imaging analysis of PKH26-labeling in hAMSCs in vivo at day 0, day 7, day 14, and day 21. **d** Immunofluorescence images of the sections from day-7, day-14, and day-21 skin tissues in PKH26-labeled hAMSCs. hAMSCs were co-stained with MAB1281 (antibody to human-specific nuclei) and CD90 and imaged by confocal microscope. **e** Representative photomicrographs of H&E stained sections from day-7 and day-14 wounds injected with PBS, hAMSCs, H-DMEM, or hAMSC-CM. The arrows indicate the layers of keratinocytes. Normal skin was used as a control

To examine the therapeutic potential of hAMSCs and hAMSC-CM on wound healing, we established a mouse deep second-degree burn injury model and injected 200 μl PBS containing 2 × 10^6^ hAMSCs, or 200 μl concentrated hAMSC-CM to heat-injured wounds created in C57BL/6 mice. Similar wounds treated with PBS or H-DMEM were used as controls. The wounds at 7 days, 14 days, and 21 days were carefully measured, showing that hAMSCs and hAMSC-CM significantly accelerated wound closure compared to PBS and H-DMEM (Fig. 2a, b). For cell-tracking purposes, hAMSCs were labeled with PKH26. Mice were anesthetized after 0 days, 7 days, 14 days, and 21 days of cell injection and visualized with a whole-body fluorescent imaging system. As shown in Fig. 2c, hAMSCs were only clearly observed in the injured skin sites but were gradually reduced at 7, 14, and 21 days after injection. To verify whether hAMSCs were differentiated into other kinds of cells in vivo, antibody to human-specific nuclei (MAB1281) was used to identify human cells in mouse skin tissues. The immunostaining analysis showed that the MAB1281-positive cells also expressed CD90, suggesting that the human cells present in the skin tissues were still hAMSCs (Fig. 2d). HE staining of wounds on day 7 showed that the number of epidermal and dermal cells significantly increased in hAMSC- and hAMSC-CM-treated mice as compared with PBS- and H-DMEM-treated mice. At 14 days post-injection, HE staining showed that wounds treated with hAMSCs and hAMSC-CM displayed more layers of keratinocytes, indicating enhanced re-epithelialization when compared with PBS- and H-DMEM-treated wounds (Fig. 2e).

**Fig. 3:**
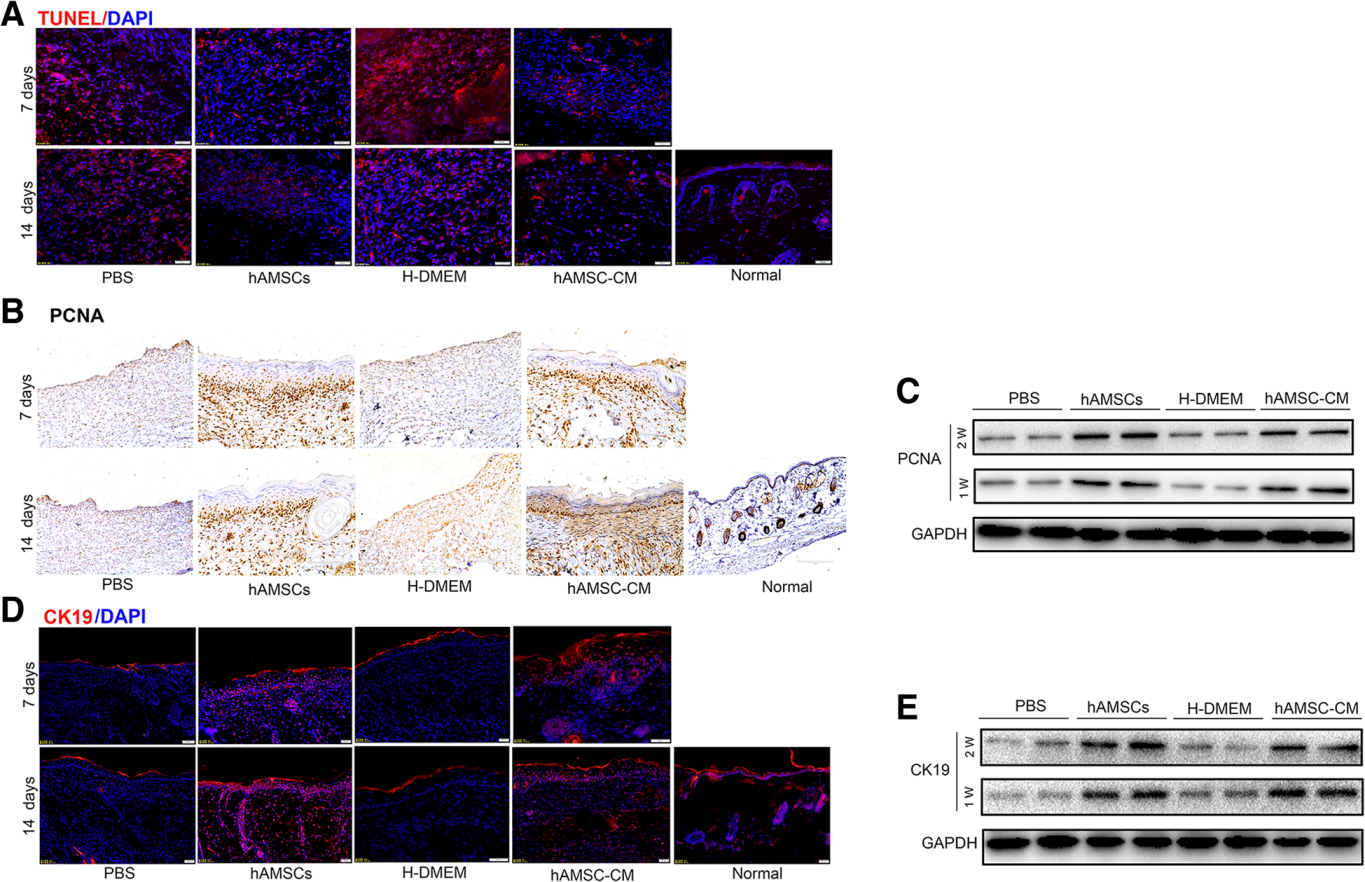
hAMSCs and hAMSC-CM inhibited heat stress-induced apoptosis and promoted proliferation of skin cells in vivo. **a** Estimation of apoptosis in sections from day-7 and day-14 skin tissues of normal, PBS, hAMSCs, H-DMEM, and hAMSC-CM groups by using the TUNEL assay. **b** Proliferation of skin cells was determined by immunohistochemistry using antibodies against PCNA in sections from day-7 and day-14 skin tissues of the PBS, hAMSCs, H-DMEM, and hAMSC-CM groups. (C) Western blot analysis of PCNA protein levels in day-7 and day-14 skin tissues of the PBS, hAMSCs, H-DMEM, and hAMSC-CM groups. **d** Representative immunofluorescence images of CK19 expression for re-epithelialization in skin tissues. **e** Western blot assay for CK19 expression in skin tissues at 7 days and 14 days after treatment

In order to test the treatment effect of hAMSCs and hAMSC-CM on apoptosis in the skin tissue, sections from normal, PBS, hAMSCs, H-DMEM, and hAMSC-CM groups were subjected to TUNEL staining. After 7 days and 14 days of injection, a significant reduction in cell apoptosis was observed in the hAMSCs and hAMSC-CM group compared to PBS and H-DMEM group, suggesting that hAMSCs and hAMSC-CM inhibited heat stress-induced apoptosis of skin cells in vivo (Fig. 3a). To delineate the pro-proliferative and pro-epithelialization effect of hAMSCs and hAMSC-CM in vivo, immunohistochemistry and western blot were used to detect the expression of PCNA and CK19 in day-7 and day-14 wounds injected with PBS, hAMSCs, or hAMSC-CM. The results showed that hAMSCs and hAMSC-CM significantly increased the expression levels of PCNA and CK19 in the hAMSC- and hAMSC-CM-injected skin tissues compared with those in the PBS- and H-DMEM-injected skin tissues (Fig. 3b–e). No significant difference in cell apoptosis and proliferation was observed in the hAMSCs group compared to the hAMSC-CM group. After 7 days and 14 days of injection, CD31 immunofluorescence staining analysis revealed that the numbers of new blood vessels in the hAMSCs and hAMSC-CM group were significantly increased compared to PBS and H-DMEM group (Additional file 2: Figure S2A), indicating that hAMSCs and hAMSC-CM promoted neovascularization in vivo*.* In vitro, tube formation assay was performed to assess the ability of hAMSC-CM to promote HUVEC angiogenesis. When compared with the control group, there were more tubular structures in the hAMSC-CM at 2 h, 4 h, and 6 h of incubation, which means hAMSC-CM continuously promoted angiogenesis (Additional file 2: Figure S2B and S2C). Taken together, these results indicate that hAMSCs and hAMSC-CM promote the repair of skin burn injury by enhancing proliferation, angiogenesis, and inhibiting apoptosis of skin cells in the wound area.

### hAMSCs and hAMSC-CM inhibited heat stress-induced apoptosis and promoted proliferation of HaCAT and DFL cells in vitro

**Fig. 4:**
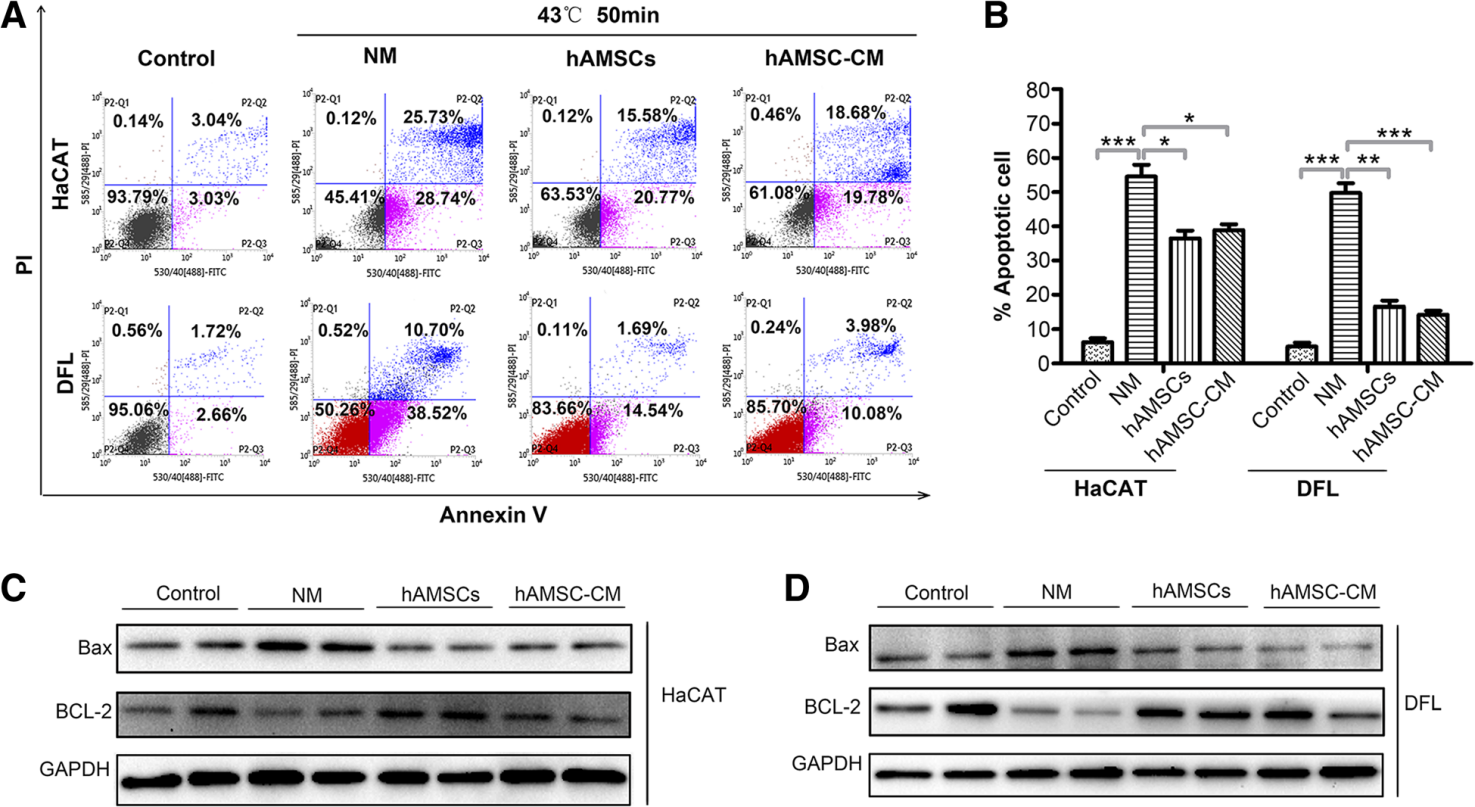
hAMSCs and hAMSC-CM inhibited heat stress-induced apoptosis of HaCAT and DFL cells. **a** Examination of apoptosis cells in HaCAT and DFL cells with normal medium (NM), hAMSCs, and hAMSC-CM after 43 °C 50 min heat stress. The apoptosis of cells was assessed by FACS after 24 h of treatment. **b** Quantitative analysis of the percentage of apoptotic cells as shown in **a** (*n* = 3). **c** The expression analysis of Bcl-2 and Bax in HaCAT cells of different groups by immunoblotting assay. **d** Western blot assay for Bcl-2 and Bax expression in DFL cells of different groups. Significance was measured using a two-way ANOVA. *P < 0.05, **P < 0.01, ***P < 0.001

To investigate the biological effect of hAMSCs and hAMSC-CM on the apoptosis and proliferation of heat stress-injured skin cells, HaCAT and DFL were treated at 43 °C for 50 min and then treated with normal medium (NM), hAMSCs (at a ratio of skin cells to hAMSCs of 1:1), or 10% hAMSC-CM (10X). HaCAT and DFL cells not treated at 43 °C for 50 min and cultured in a normal medium were used as control. Flow cytometry analysis was performed after 24 h of treatment. As shown in Fig. 4a, the apoptotic rates of HaCAT and DFL cells co-cultured with hAMSCs or cultured with hAMSC-CM were significantly decreased in comparison to that of the NM group. The apoptotic rates of these HaCAT cells were 36.47 ± 3.27%, 38.92 ± 2.45%, and 54.59 ± 4.90%, respectively. The apoptotic rates of DLF cells in the hAMSCs group, hAMSC-CM group, and NM group were 16.54 ± 2.45%, 24.3 ± 1.63%, and 49.74 ± 4.08%, respectively (Fig. 4b). Additionally, the apoptotic associated proteins Bcl-2 and Bax in HaCAT and DFL cells were detected by western blot. When compared with NM group, Bcl-2 levels were increased and Bax levels were decreased in the hAMSCs and hAMSC-CM group (Fig. 4c, d).

**Fig. 5:**
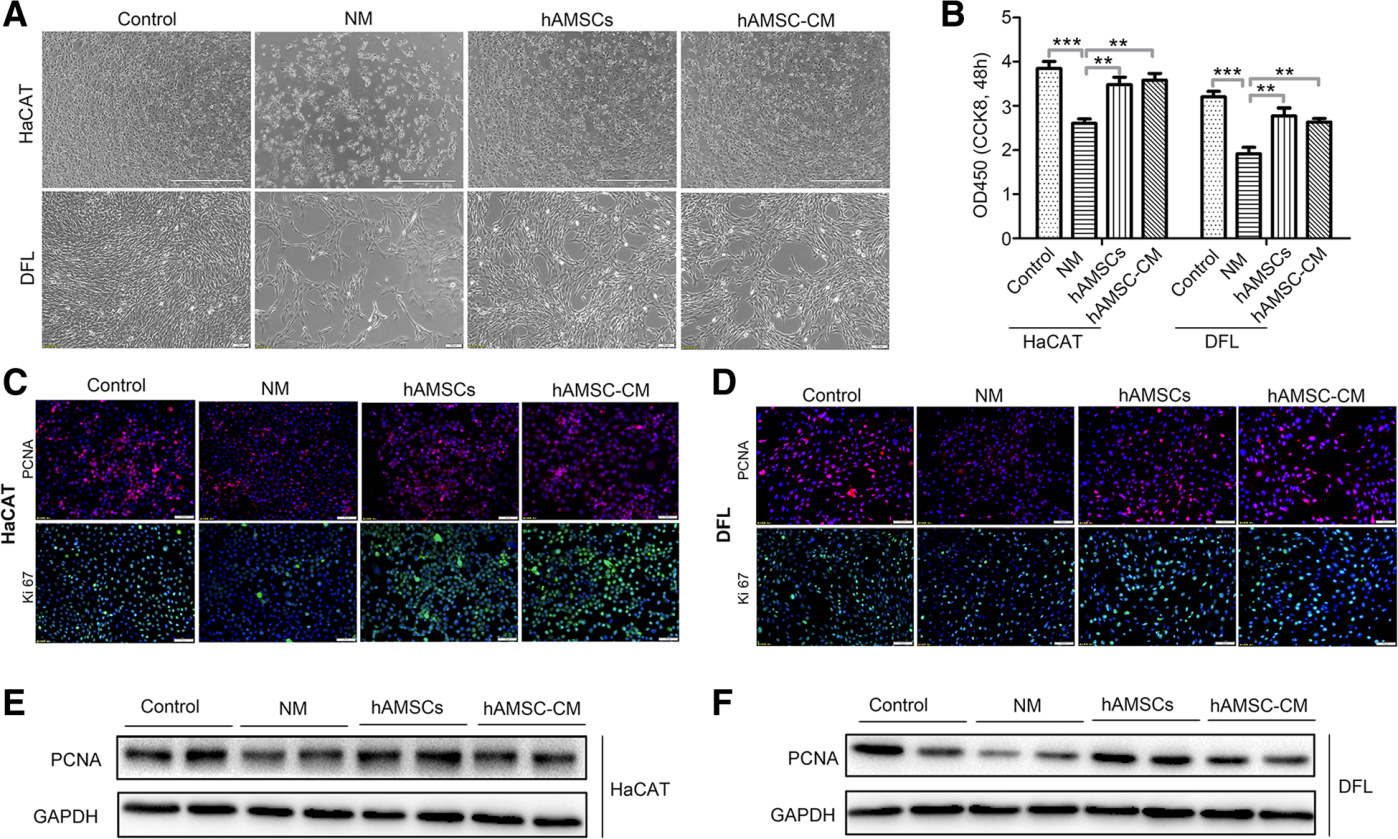
hAMSCs and hAMSC-CM inhibited heat stress-induced growth inhibition of HaCAT and DFL cells and promoted their proliferation. **a** Representative images of the cell proliferation assay treated with normal medium (NM), hAMSCs, and hAMSC-CM for 48 h after 43 °C 50 min heat stress. **b** Cell viability for 48 h after treatment with normal medium, hAMSCs, and hAMSC-CM using a CCK-8 assay. **c** The expression levels of PCNA and Ki67 in HaCAT cells of different groups by immunofluorescence staining. **d** Immunofluorescence staining for determining the expression levels of PCNA and Ki67 in DFL cells of different groups. **e** Western blot assay for PCNA expression in HaCAT cells treated with normal medium, hAMSCs, and hAMSC-CM for 48 h after 43 °C 50 min heat stress. **f** Western blot assay for PCNA expression in DFL cells treated with normal medium, hAMSCs, and hAMSC-CM for 48 h after 43 °C 50 min heat stress. HaCAT and DFL cells not treated at 43 °C for 50 min and cultured in normal medium were used as controls. Significance was measured using a two-way ANOVA. *P < 0.05, **P < 0.01, ***P < 0.001

CCK8 assay showed that the numbers of HaCAT and DFL cells in the hAMSCs and hAMSC-CM groups were sharply increased when compared with the NM group, indicating that significant vitality promotion in HaCAT and DFL cells was induced by hAMSCs and hAMSC-CM at 48 h. In contrast, there was no obvious difference between the hAMSCs group and hAMSC-CM group (Fig. 5a, b). We further studied the expression of proliferation-related proteins in control, heat, hAMSCs, and hAMSC-CM group by using immunofluorescent staining and western blot. As shown in Fig. 5c–f, the expression levels of proliferation-related proteins Ki67 and PCNA in HaCAT and DFL cells of the hAMSCs and hAMSC-CM groups were significantly higher than that in the NM group. These results demonstrated that hAMSCs inhibited heat stress-induced apoptosis in skin cells and promoted their proliferation in a paracrine manner.

### hAMSCs and hAMSC-CM promoted heat-injured skin cell migration and wound closure

**Fig. 6:**
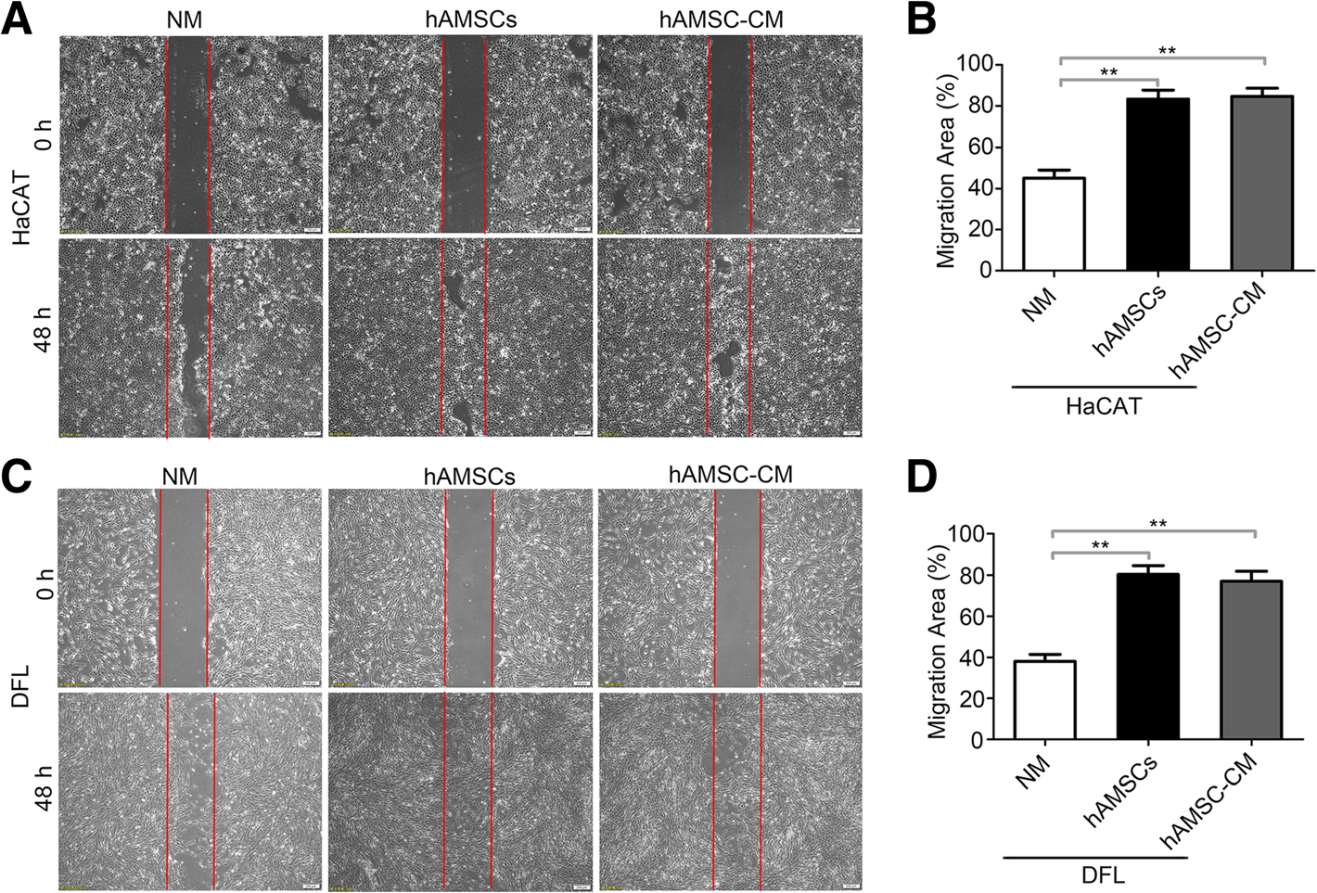
hAMSCs and hAMSC-CM promoted the migration and wound closure of heat stress-injured HaCAT and DFL cells in a wound scratch assay. Representative images of HaCAT (**a**) and DFL (**c**) scratch assays. The images were taken immediately after the scratches had been made and then after 48 h in the presence of hAMSCs and hAMSC-CM versus normal medium (NM). The red line indicates the initiatory areas without migrating cells. Quantitative analysis of the migration area was performed for HaCAT (**b**) and DFL (**d**), respectively. The in vitro wound-healing assay showed that hAMSCs and hAMSC-CM strongly improved the HaCAT and DFL wound closure compared with the NM group. Significance was measured using a two-way ANOVA. *P < 0.05, *P < 0.01, ***P < 0.001

To mimic wound healing in vitro and examine whether cytokines secreted from hAMSCs promote cell migration during wound closure, confluent HaCAT and DFL cells were treated at 43 °C for 50 min and were scratched to create a linear wound. HaCAT and DFL cells were then cultured with normal medium, hAMSCs, and hAMSC-CM, respectively. The wound margin was marked on the picture, and the wound healing rate was quantified after 2 days of culture. The results showed that higher HaCAT and DFL cell migration was observed in the presence of hAMSCs and hAMSC-CM as compared to the normal medium. At 48 h, the wound closure of HaCAT scratch assays was 83.33 ± 6.24% in the hAMSCs group and 84.67 ± 5.56% in the hAMSC-CM group, whereas in the NM group, it was 45.0 ± 5.72%. Similarly, the wound closure of DFL scratch assays was 80.28 ± 5.76% in the hAMSCs group, 77 ± 6.58% in the hAMSC-CM group, and 38 ± 4.90% in the PBS group. However, no significant difference between the hAMSCs group and the hAMSC-CM group was observed (Fig. 6a–d). The scratch wound assay demonstrated that hAMSCs and hAMSC-CM could significantly increase the rate of heat-injured skin cell wound closure compared with a normal medium in vitro.

### hAMSCs and hAMSC-CM activate PI3K/AKT/mTOR and GSK3β/β-catenin pathway in heat-injured skin cells

**Fig. 7:**
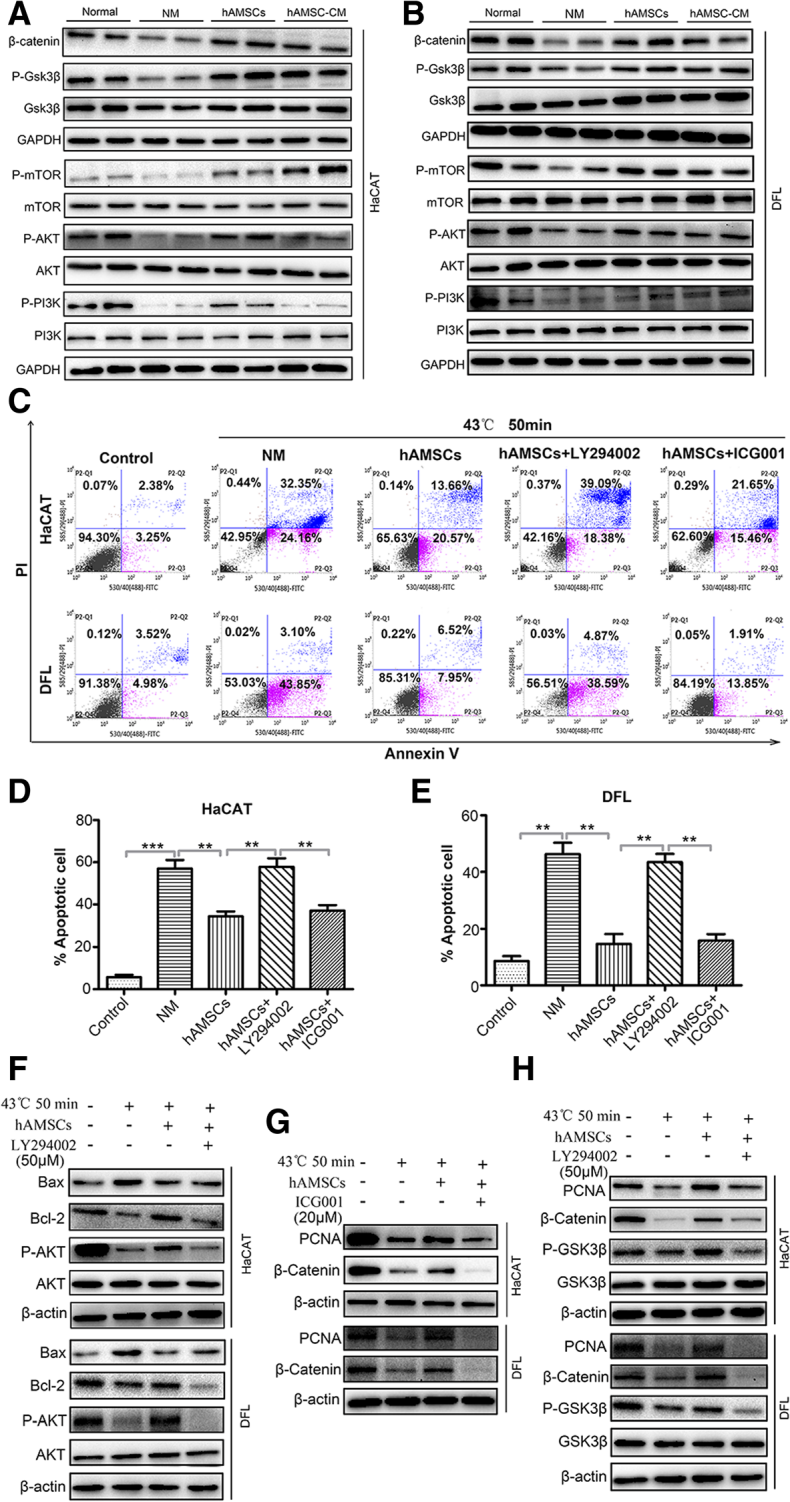
hAMSCs and hAMSC-CM inhibited heat stress-induced apoptosis and promoted proliferation of heat-injured HaCAT and DFL cells by activating PI3K/AKT signaling. The phospho-PI3K, phospho-AKT, phospho-mTOR, phospho-Gsk3β, and β-catenin were determined by western blot analysis in heat-injured HaCAT (**a**) and DFL (**b**) cells. The downregulations of the phosphorylation of the protein were prevented by hAMSCs and hAMSC-CM. **c** Apoptosis analysis of HaCAT and DFL cells. The cells were subjected to heat stress (43 °C, 50 min) and treated with normal medium (NM), hAMSCs, or hAMSCs with LY294002 (50 μM) or ICG001 (20 μM). The apoptosis of cells was assessed by FACS after 24 h of treatment, and the percentage of apoptotic HaCAT (**d**) and DFL (**e**) cells was quantitatively analyzed as shown in **c** (*n* = 3). **f** Western blot assay for Bax, Bcl-2, P-AKT, and AKT in heat-injured HaCAT and DFL cells treated with hAMSCs in the presence or absence of LY294002 (50 μM). **g** Western blot assay for β-catenin and PCNA in heat-injured HaCAT and DFL cells treated with hAMSCs in the presence or absence of ICG001 (20 μM). **h** Western blot assay for Gsk3β, P- Gsk3β, β-catenin, and PCNA in heat-injured HaCAT and DFL cells treated with hAMSCs in the presence or absence of LY294002 (50 μM). HaCAT and DFL cells not treated at 43 °C for 50 min and cultured in a normal medium were used as controls. Significance was measured using a two-way ANOVA. *P < 0.05, *P < 0.01, ***P < 0.001

Since we found that hAMSCs and hAMSC-CM inhibited heat stress-induced apoptosis and promoted proliferation of HaCAT and DFL cells in vivo and in vitro, we further investigated its mechanisms. Since PI3K/AKT [25] and GSK3β/β-catenin [26, 27] signaling pathways play important roles in cell survival, cell apoptosis, and skin development, we hypothesized that PI3K/AKT and GSK3β/β-catenin signaling might be involved in the biological effects of hAMSCs and hAMSC-CM on the heat-injured skin cells. As expected, western blot analysis showed that levels of phospho-PI3K, phospho-AKT, phospho-mTOR, phospho-Gsk3β, and β-catenin were decreased in heat stress-injured HaCAT and DFL cells compared to normal-treated controls. Importantly, this effect was prevented with hAMSCs and hAMSC-CM in both HaCAT and DFL cells after heat stress injury (Fig. 7a, b). Then to elucidate if the PI3K/AKT signaling pathway is necessary for the hAMSC-mediated apoptosis inhibition, we treated the heat-injured HaCAT and DFL cells with hAMSCs with or without PI3K inhibitor, LY294002. We found that hAMSC treatment reduced the numbers of apoptosis cells while the simultaneous treatment with LY294002 reversed this effect. We also treated the heat-injured HaCAT and DFL cells with hAMSCs with or without β-catenin inhibitor, ICG001. In contrast, we found that inhibition of β-catenin with ICG001 could not reverse the effects of hAMSCs on the apoptosis inhibition (Fig. 7c–e). Western blot analysis showed that hAMSCs induced the activation of AKT which led to the increase of Bcl-2 protein level and the decline of Bax protein level, which were suppressed by LY294002 (Fig. 7f). These results indicated that hAMSCs reversed acute heat stress-induced apoptosis in HaCAT and DFL cells through activation of PI3K/AKT signaling pathway.

Next, the role of β-catenin and AKT activation in hAMSC-mediated promotion of cell proliferation was examined. As shown in Fig. 7g, ICG001 significantly inhibited the increase of β-catenin and PCNA expression by hAMSCs. This result revealed that hAMSCs promoted the proliferation of heat-injured HaCAT and DFL cells by increasing the expression of β-catenin. Considering that PI3K/AKT and GSK3β/β-catenin were both activated by hAMSCs, we investigated the relationship between PI3K/AKT and GSK3β/β-catenin after hAMSC treatment. Inhibition of AKT by LY294002 significantly inhibited the phosphorylation of GSK3β and the increase of β-catenin and PCNA expression, suggesting that hAMSCs mediate the activation of GSK3β/β-catenin signaling dependent of PI3K/AKT signaling (Fig. 7h).

### A panel of cytokines from hAMSCs might contribute to the acceleration of the thermal burn wound healing

**Fig. 8:**
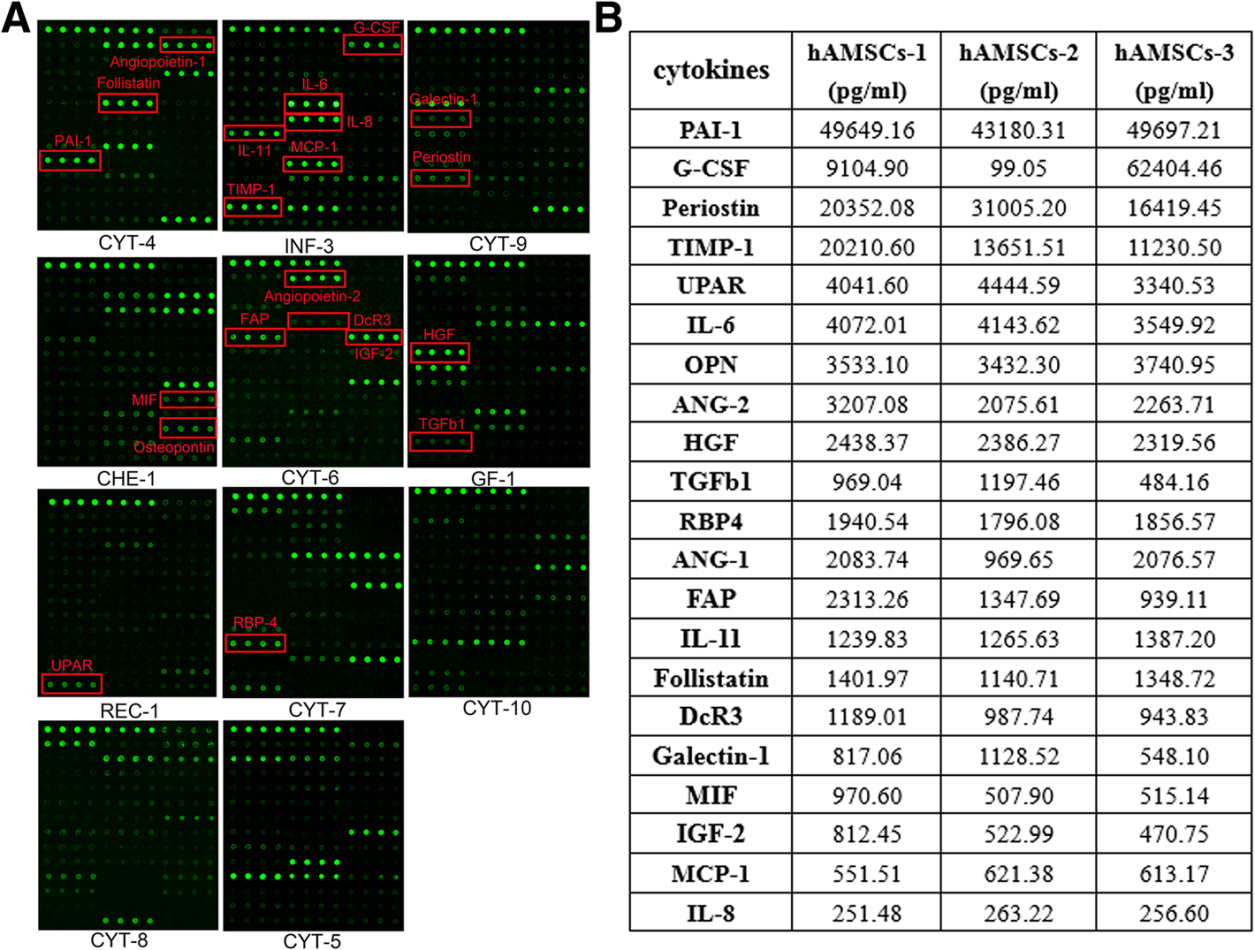
Cytokine expression profile of hAMSCs. The cytokine expression profile in the conditioned media collected from hAMSCs was detected using the RayBio Human Cytokine Antibody Array 440, which allows the detection of 440 cytokines in one experiment. **a** The representative image of cytokine antibody array. The cytokines in the hAMSC-CM which may activate PI3K/AKT signaling are highlighted with red boxes. **b** The concentration of cytokines of interest in each hAMSC-CM sample is shown. Values shown are obtained from standard curve generated against the various proteins, *n* = 4

Since hAMSCs co-culture and hAMSC-CM inhibited heat stress-induced apoptosis and promoted proliferation of HaCAT and DFL cells, we therefore hypothesized that the observed anti-apoptosis and pro-proliferation effects were mediated by the paracrine secretion of soluble factors from hAMSCs. To identify the cytokines secreted by hAMSCs that may activate PI3K/AKT signaling, the cytokine profiles of cell supernatants from hAMSCs were analyzed using a RayBiotech Human Cytokine Antibody Array. Among 440 cytokines evaluated (Fig. 8a), some cytokines were not detectably or showed extremely low expression (data not shown). Approximately 200 secreted factors were detected, and the cytokines with an average concentration higher than 200 pg/ml are shown in Additional file 3: Table S1. Our results showed that hAMSCs expressed high levels of PAI-1 [28], C-GSF [29, 30], periostin [31–33], and TIMP-1 [34], which have been proved to accelerate wound healing and increase the anti-apoptotic and proliferative capacity of skin cells by upregulating PI3K/AKT signaling pathway. The concentration of these cytokines was 47,508.89 pg/ml, 23,869.67 pg/ml, 22,592.24 pg/ml, and 11,697.53 pg/ml, respectively. We also found that uPAR [35–38], IL-6 [39], osteopontin (OPN) [40–42], Angiopoietin-2 (ANG-2) [43], HGF [44–46], TGFb1 [47, 48], RBP4 [49], Angiopoietin-1 (ANG-1) [50–52], FAP [53], IL-11 [54, 55], Follistatin [56], DcR3 [57], Galectin-1 [58], MIF [59, 60], IGF-2 [61, 62], MCP-1 [63], and IL-8 [64, 65] were moderately secreted by hAMSCs, which may activate AKT signaling (Fig. 8a, b).

## Discussion

Stem cells have been considered a promising source of seed cells for biological therapeutics and tissue engineering. However, in terms of clinical applications, the safety, immunological rejection, and ethical criteria are strictly required. The amniotic membrane, a medical waste after birth, has been reported to contain a population of multipotent stem cells exhibiting characteristics of MSCs. However, less effort has been made on hAMSCs. In the present study, we report that hAMSCs can be easily isolated from the donor’s amnion without ethical concerns and showed fibroblast-like morphology. hAMSCs express numerous markers such as the core pluripotency genes (OCT4, SSEA-4, and Nanog) and MSC-specific surface markers (CD29, CD73, CD105, and CD29), but the absence of hematopoietic markers (CD133, CD34, CD45) and HLA Class II (HLA-DR). Our results have also shown that the hAMSCs have low expression of HLA Class I (HLA-ABC) but none of its co-stimulatory molecules CD80, CD86, and CD40, suggesting that there are a weak immunogenicity and potential immune tolerance after transplantation of hAMSCs. In addition, we also found that hAMSCs have multi-lineage differentiation potentials and no tumorigenicity both in vivo and in vitro. These characteristics make hAMSCs as a promising source of stem cells for clinical application.

Several studies showed that MSCs derived from different tissues, such as bone marrow (BM) [14, 20], umbilical cord (UC) [15], and adipose [66], are capable of enhancing and improving wound healing in vivo and in vitro. Interestingly, it has been well-documented that the paracrine factors such as growth factors, cytokines, and exosome of stem cells contribute to the therapeutic effect [67, 68]. Anna et al. demonstrated that UC-derived MSCs enhanced normal skin fibroblast proliferation and migration and promoted wound healing in an excisional full-thickness skin murine model through paracrine signaling [15]. Zhang et al. found that MSC-derived exosome promoted proliferation and inhibited apoptosis of skin cells after heat stress in vitro [17]. In the present study, we reported that hAMSCs and hAMSCs-CM significantly promoted thermal burn wound healing. When the skin on the back of mice is injured with 80 °C water for 100 s, the skin cells will undergo apoptosis in a short time (within a few days). Our study showed that in vivo transplantation of hAMSCs and hAMSC-CM significantly enhanced re-epithelialization and accelerated wound closure by inhibiting apoptosis and enhancing proliferation of heat-injured skin cells in the wound area in vivo*.* However, hAMSCs and hAMSC-CM had no significant differences in the wound healing. Therefore, we believe that the critical period of hAMSCs and hAMSC-CM in the treatment of skin scald is the first few days after injury. Thus, despite the limited duration of the action of hAMSC-CM, it still has a similar therapeutic effect as hAMSCs. Although our results showed that hAMSCs could still be found in heat-injured skin wounds 14 and 21 days after cell injection, the numbers of hAMSCs on day 14 or 21 were significantly reduced compared with that of day 7 (Fig. 2c), indicating that the secretion of cytokines might be also significantly reduced and their concentration might be insufficient to significantly promote skin cell proliferation and wound injury repair. Meanwhile, we also demonstrated that hAMSCs and hAMSCs-CM inhibited heat stress-induced apoptosis in skin cells, promoted their proliferation, and increased their migration in a paracrine manner in vitro. To our knowledge, this is the first time to report that hAMSCs and hAMSC-derived factors have the ability to inhibit heat stress-induced apoptosis of HaCAT and DFL cells and promoted proliferation and accelerated wound closure in vitro and in vivo.

The serine/threonine kinase AKT is an important component of the PI3K signaling pathway; active AKT controls many cellular functions, including cell growth, survival, and cell metabolism [69]. Wnt/β-catenin signaling plays an important role in embryonic patterning, cell proliferation, differentiation, and angiogenesis [70]. Axin serves as a scaffold protein to recruit GSK3β and CKIα (caspase kinase alpha) along with APC to form a complex with beta-catenin, resulting in beta-catenin phosphorylation, ultimately causing its degradation [71]. The phosphorylation of GSK3β is a classic negative regulator of Wnt signaling pathway. Many investigators have described that PI3K/AKT and GSK3β/β-catenin signaling play a key role in skin development and cutaneous wound healing [26, 33, 72]. Our results show that hAMSCs and hAMSC-CM significantly reversed heat stress-induced decline of phosphorylation of PI3K, AKT, GSK3β, and β-catenin expression levels in HaCAT and DFL cells, indicating that activations of PI3K/AKT and GSK3β/β-catenin signaling pathways might be involved in the therapeutic effects of hAMSCs and hAMSC-CM on the heat-injured wound healing in skin cells. Furthermore, we demonstrated that hAMSC- or hAMSC-CM-mediated activation of GSK3β/β-catenin signaling was dependent on PI3K/AKT signaling since the inhibition of hAMSCs or CM on apoptosis could be reversed by PI3K inhibitor LY294002, but not β-catenin inhibitor ICG001 although ICG001 significantly inhibited the hAMSC-mediated increase of PCNA expression. All of these results indicated that hAMSCs and hAMSC-CM reversed acute thermal injury-induced apoptosis and growth inhibition in skin cells through activation of PI3K/AKT signaling pathway.

To identify the hAMSC-secreted molecules involved in the activation of PI3K/AKT pathway, the cytokines of hAMSC-CM were analyzed using an antibody array. Our results showed that hAMSC-CM contained high levels of PAI-1, C-GSF, periostin, and TIMP-1. PAI-1 is the most abundant factor in the hAMSC-CM. Harman et al. found that MSC-derived PAI-1 significantly increased DFL migration in vitro and improved wound healing in vivo by decreasing time to wound closure [73]. Lademann et al. found PAI-1 protects fibrosarcoma cells from etoposide-induced apoptosis through activation of PI3K/AKT cell survival pathway [28]. G-CSF, a hematopoietic cytokine and potent stem cell mobilization agent, has been proved to accelerate wound healing by enhancing angiogenesis and attenuating apoptosis [30], and PI3K/AKT signaling pathway would be activated in response to G-CSF stimulation [29]. Periostin, one of the matricellular proteins, is normally expressed in adult skin, which is highly upregulated during wound healing [31]. Periostin has the ability to activate the PI3K/AKT signaling pathway in tumor cells by interacting with integrin molecules [74]. Recently, increasing evidence suggested that periostin is capable of dramatically increasing the migratory and proliferative abilities of epithelial cells and dermal fibroblasts by upregulating AKT/mTOR signaling pathway [32, 33]. Tissue inhibitor of metalloproteinases-1 (TIMP-1) possesses actions of promoting growth and anti-apoptosis in cells. It has been reported that TIMP- 1 reduced cell apoptosis during the process of wound healing [34]. In addition, other cytokines such as uPAR, IL-6, OPN, ANG-2, HGF, TGFb1, RBP4, ANG-1, FAP, IL-11, Follistatin, DcR3, Galectin-1, MIF, IGF-2, MCP-1, and IL-8 were moderately secreted by hAMSCs, and the cytokines have been identified to have the ability to activate PI3K/AKT pathway. However, although the acceleration of hAMSCs and hAMSC-CM on skin wound healing may be involved in activating PI3K/AKT pathway, it is hard to determine which cytokine(s) in hAMSC-CM mediate the activation of PI3K/AKT pathway due to the complexity of hAMSC-CM components.

## Conclusion

In the present study, we demonstrated that hAMSCs and hAMSC-CM accelerate skin wound healing in vivo and inhibit acute heat stress-induced skin cells apoptosis and promoted their proliferation in vitro via activation of PI3K/AKT pathway. Antibody Array assay showed that PAI-1, C-GSF, periostin, TIMP-1, and uPAR secreted by hAMSCs might be involved in the activation of PI3K/AKT signaling pathway. Our findings suggest that the administration of hAMSCs or hAMSC-CM may be a novel therapeutic strategy for skin injury repair clinically.

## Additional files

Additional file 1: **Figure S1.** Tumorogenesis of hAMSCs in vivo and in vitro. (A) hAMSCs and HepG2 cells were grown in soft agar, and the colony formation was analyzed after 30 days of cell growth. (B) 5 × 10^6^ hAMSCs cells were injected into the right back and left thigh muscle of NOD-SCID mice for observation of teratoma formation. There was no any tumor formation after 5 months of hAMSC injections. Embryonic stem cells were used as a positive control. (TIF 8547 kb)

Additional file 2: **Figure S2.** hAMSCs and hAMSC-CM enhanced neovascularization in vivo and induce HUVECs angiogenesis in vitro. (A) Representative immunofluorescence images of CD31 expression in the wound area after treatment with PBS, hAMSCs, H-DMEM, and hAMSC-CM for 7 days and 14 days. Normal skin was used as a control. (B) Enhanced tube formation in HUVECs treated with hAMSC-CM at different time point. (TIF 5037 kb)

Additional file 3: **Table S1.** Antibody array assay for determining secretions of the cytokines from hAMSCs. (DOCX 16 kb)

## Data Availability

The data that support the findings of this study are available from the corresponding author upon reasonable request.

## Abbreviations

ANG: Angiopoietin
BM: Bone marrow
CM: Conditioned medium
DFL: Dermal fibroblasts
FACS: Fluorescence-activated cell sorting
hAESCs: Human amniotic epithelial stem cells
hAMSCs: Human amniotic mesenchymal stem cells
H-DMEM: High-glucose Dulbecco’s modified Eagle’s medium
HRP: Horseradish peroxidase
MSCs: Mesenchymal stem cells
NM: Normal medium
OPN: Osteopontin
PE: Phycoerythrin
PI: Propidium iodide
RT-PCR: Reverse transcription-polymerase chain reaction
UC: Umbilical cord
