# Supplementary figures and images for: Human amniotic mesenchymal stem cells and their paracrine factors promote wound healing by inhibiting heat stress-induced skin cell apoptosis and enhancing their proliferation through activating PI3K/AKT signaling pathway
Source: Stem Cell Res Ther. 2019 Aug 9;10:247. doi: 10.1186/s13287-019-1366-y (PMC6688220; doi:10.1186/s13287-019-1366-y)

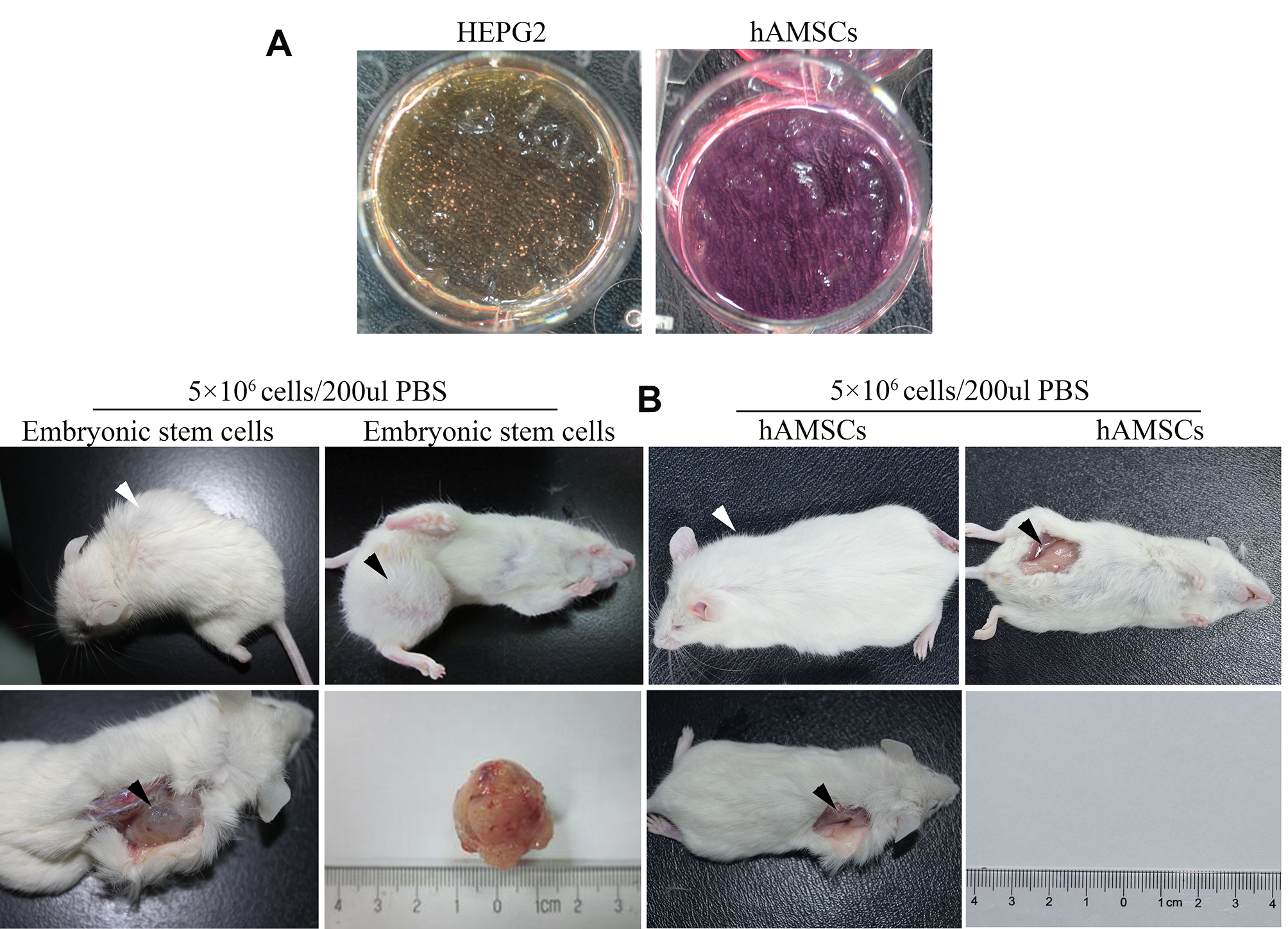

Supplement: Supplementary file 1 — Figure S1. Tumorogenesis of hAMSCs in vivo and in vitro. (A) hAMSCs and HepG2 cells were grown in soft agar, and the colony formation was analyzed after 30 days of cell growth. (B) 5 × 10^6^ hAMSCs cells were injected into the right back and left thigh muscle of NOD-SCID mice for observation of teratoma formation. There was no any tumor formation after 5 months of hAMSC injections. Embryonic stem cells were used as a positive control. (TIF 8547 kb) [file 13287_2019_1366_MOESM1_ESM.tif]

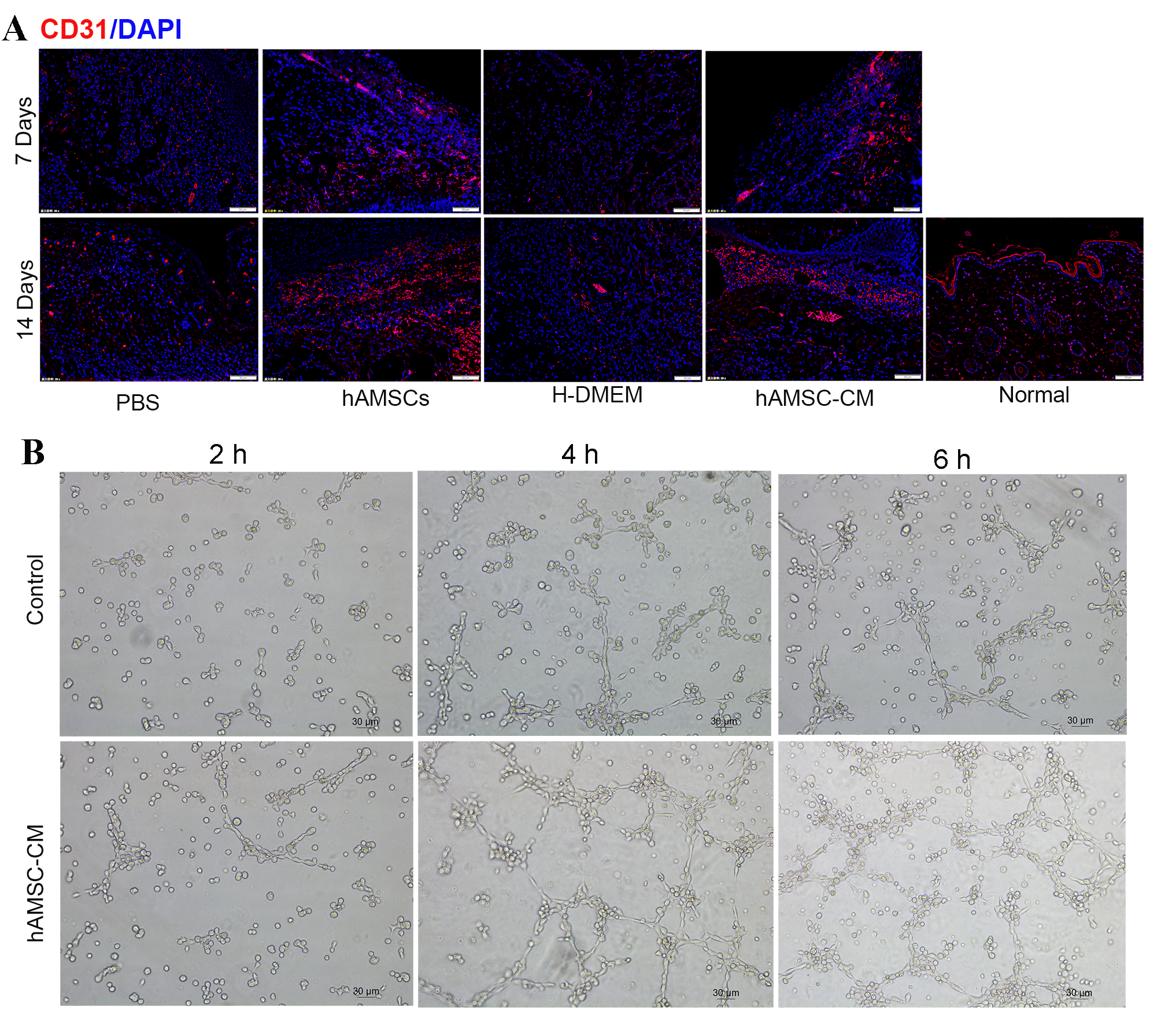

Supplement: Supplementary file 2 — Figure S2. hAMSCs and hAMSC-CM enhanced neovascularization in vivo and induce HUVECs angiogenesis in vitro. (A) Representative immunofluorescence images of CD31 expression in the wound area after treatment with PBS, hAMSCs, H-DMEM, and hAMSC-CM for 7 days and 14 days. Normal skin was used as a control. (B) Enhanced tube formation in HUVECs treated with hAMSC-CM at different time point. (TIF 5037 kb) [file 13287_2019_1366_MOESM2_ESM.tif]
