# Supplementary material for: Human amniotic mesenchymal stem cells and their paracrine factors promote wound healing by inhibiting heat stress-induced skin cell apoptosis and enhancing their proliferation through activating PI3K/AKT signaling pathway
Source: Stem Cell Res Ther. 2019 Aug 9;10:247. doi: 10.1186/s13287-019-1366-y (PMC6688220; doi:10.1186/s13287-019-1366-y)
Supplement: Supplementary file 3 — Table S1. Antibody array assay for determining secretions of the cytokines from hAMSCs. (DOCX 16 kb) [file 13287_2019_1366_MOESM3_ESM.docx]

| **cytokines** | **Concentration**  **(pg/ml)** | **cytokines** | **Concentration**  **(pg/ml)** | **cytokines** | **Concentration**  **(pg/ml)** |
| --- | --- | --- | --- | --- | --- |
| **PAI-1** | 47508.89 | **LAP(TGFb1)** | 2133.15 | **TLR4** | 628.21 |
| **TSP-1** | 46888.60 | **GCP-2** | 1984.95 | **CXCL16** | 611.10 |
| **IGFBP-3** | 45401.20 | **IGFBP-4** | 1964.50 | **IGF-2** | 602.06 |
| **VEGF R1** | 44504.73 | **MMP-2** | 1893.39 | **ACE-2** | 598.81 |
| **Dkk-3** | 37200.47 | **RBP4** | 1864.39 | **MCP-1** | 595.35 |
| **ANGPTL4** | 28309.84 | **ANG-1** | 1709.99 | **MCP-3** | 577.51 |
| **G-CSF** | 23869.67 | **FAP** | 1533.35 | **DPPIV** | 551.44 |
| **Periostin** | 22592.24 | **ADAMTS13** | 1514.22 | **DR3** | 545.65 |
| **Nidogen-1** | 19445.91 | **Thyroglobulin** | 1430.57 | **IL-21** | 542.56 |
| **Thrombospondin-2** | 19240.45 | **CD48** | 1401.87 | **Lymphotactin** | 532.83 |
| **NCAM-1** | 16793.46 | **Galectin-3** | 1383.17 | **APRIL** | 520.30 |
| **CHI3L1** | 11930.42 | **Activin A** | 1357.88 | **Mer** | 453.28 |
| **DKK-1** | 11930.42 | **LRIG3** | 1321.34 | **Bfgf** | 423.05 |
| **TIMP-1** | 11697.53 | **Cystatin B** | 1310.28 | **ICAM-1** | 410.39 |
| **Albumin** | 11391.93 | **IL-11** | 1297.52 | **Ck beta 8-1** | 406.86 |
| **TIMP-2** | 9685.84 | **Follistatin** | 1297.13 | **SCF R** | 402.80 |
| **GROa** | 9143.29 | **TFPI** | 1248.83 | **IL-1 F8** | 399.34 |
| **bIG-H3** | 9048.11 | **NSE** | 1201.45 | **Syndecan-1** | 382.94 |
| **B2M** | 8007.65 | **CTLA4** | 1143.79 | **TACE** | 382.33 |
| **AMICA** | 7198.75 | **uPA** | 1128.00 | **CEACAM-5** | 374.70 |
| **IGFBP-6** | 7002.61 | **gp130** | 1057.82 | **Angiogenin** | 364.30 |
| **Decorin** | 6627.55 | **DcR3** | 1040.19 | **Marapsin** | 357.57 |
| **Ferritin** | 6498.56 | **MMP-3** | 978.35 | **EMMPRIN** | 347.31 |
| **Legumain** | 5166.15 | **Cadherin-4** | 958.98 | **Fetuin A** | 316.81 |
| **FLRG** | 4881.14 | **GASP-1** | 947.92 | **Fractalkine** | 314.59 |
| **ENA-78** | 4644.60 | **MMP-10** | 930.41 | **BMP-5** | 308.19 |
| **sFRP-3** | 4568.18 | **ADAM9** | 918.46 | **IL-1 F9** | 303.67 |
| **MMP-1** | 4346.03 | **TGFb1** | 883.53 | **DNAM-1** | 282.60 |
| **PF4** | 4101.66 | **FGF-7** | 865.68 | **IL-27** | 271.78 |
| **uPAR** | 3931.90 | **Cystatin C** | 855.60 | **Galectin-9** | 269.88 |
| **IL-6** | 3921.84 | **BCAM** | 851.75 | **Procalcitonin** | 266.46 |
| **Pentraxin 3** | 3614.37 | **Galectin-1** | 831.22 | **Layilin** | 265.98 |
| **OPN** | 3568.78 | **Midkine** | 774.61 | **IL-8** | 257.09 |
| **Prolactin** | 3325.15 | **CA125** | 729.71 | **IL-20** | 218.19 |
| **Cathepsin B** | 2959.60 | **Galectin-2** | 716.10 | **IL-31** | 217.84 |
| **CA19-9** | 2881.31 | **Follistatin-like 1** | 711.14 | **IL-23** | 205.04 |
| **ANG-2** | 2515.46 | **MIF** | 664.54 | **BMP-7** | 204.06 |
| **HGF** | 2381.40 | **Angiostatin** | 659.75 |  |  |
| **GRO** | 2214.43 | **IL-17F** | 654.16 |  |  |
